# Comparative Study on Conductive Knitted Fabric Electrodes for Long-Term Electrocardiography Monitoring: Silver-Plated and PEDOT:PSS Coated Fabrics

**DOI:** 10.3390/s18113890

**Published:** 2018-11-12

**Authors:** Amale Ankhili, Xuyuan Tao, Cédric Cochrane, Vladan Koncar, David Coulon, Jean-Michel Tarlet

**Affiliations:** 1 École Nationale Supérieure des Arts et Industries Textiles/Génie et Matériaux Textiles Laboratory (ENSAIT/GEMTEX), 2 Allée Louis et Victor Champier, F-59100 Roubaix, France; xuyuan.tao@ensait.fr (X.T.); cedric.cochrane@ensait.fr (C.C.); vladan.koncar@ensait.fr (V.K.); 2 GEMTEX, University of Lille, Cité Scientifique, F-59650 Villeneuve d’Ascq, France; 3 @Health, Europarc de Pichaury, 1330 Rue Jean René Guillibert Gauthier de la Lauzière, F-13290 Aix-en-Provence, France; dcoulon@healthcardionexion.com (D.C.); jmtarlet@healthcardionexion.com (J.-M.T.); 4 Institution Centre de Cardiologie, 32 Bd du Roy René, 13100 Aix-En-Provence, France

**Keywords:** textiles electrodes, electrocardiography, signal quality, silver-plated electrodes, washability

## Abstract

Long-term monitoring of the electrical activity of the heart helps to detect the presence of potential dysfunctions, enabling the diagnosis of a wide range of cardiac pathologies. However, standard electrodes used for electrocardiogram (ECG) acquisition are not fully integrated into garments, and generally need to be used with a gel to improve contact resistance. This article is focused on the development of washable screen-printed cotton, with and without Lycra, textile electrodes providing a medical quality ECG signal to be used for long-term electrocardiography measurements. Several samples with different Poly(3,4-ethylenedioxythiophene):poly(styrene sulfonate) (PEDOT:PSS) concentrations were investigated. Silver-plated knitted fabric electrodes were also used for comparison, within the same process of ECG signal recording. The acquisition of ECG signals carried out by a portable medical device and a low-coast Arduino-based device on one female subject in a sitting position. Three textile electrodes were placed on the right and left forearms and a ground electrode was placed on the right ankle of a healthy female subject. Plastic clamps were applied to maintain electrodes on the skin. The results obtained with PEDOT:PSS used for electrodes fabrication have been presented, considering the optimal concentration required for medical ECG quality and capacity to sustain up to 50 washing cycles. All the ECG signals acquired and recorded, using PEDOT:PSS and silver-plated electrodes, have been reviewed by a cardiologist in order to validate their quality required for accurate diagnosis.

## 1. Introduction

The electrocardiogram (ECG) is a device for recording the heart’s electrical activity. It outputs heartbeats and the intervals between them. Physicians use an electrocardiogram to look for patterns among these heartbeats and rhythms to diagnose various heart conditions [1]. ECGs are used to measure the rate and regularity of heartbeats, the size and position of the chambers, the presence of any damage to the heart, and the effects of drugs or devices used to regulate the heart, such as artificial pacemakers [2].

To obtain permanent and accurate high-quality ECG signals, and to ensure high comfort for the user, proper electrodes are required. These electrodes should guarantee a permanent contact with the skin, optimal reliability, washability, and acceptable lifetime. Wet electrodes are already widely used for ECG monitoring. They consist of a metal snap, a silver/silver chloride (Ag/AgCl) coated sensors, a conductive hydrogel, and an adhesive foam. The hydrogel offers a stable contact interface, and therefore, decreases skin–electrode contact impedance. However, these electrodes cannot be used for long-term monitoring because the hydrogel can provoke irritation after prolonged contact with the skin. Furthermore, signals are degraded due to the gel drying out, and removal is also painful [3,4]. As a result, this type of electrode can only be used for short-term diagnostic recordings, such as taking a clinical electrocardiogram in the hospital.

For long-term ECG monitoring, conductive textiles exhibit growing potential in wearable electronics. Textile substrates intended to be used within underwear are expected to be lightweight, flexible, stretchable, conformable, washable, and long-lasting [5,6]. Conducting textiles can be made with several materials using different fabrication methods. The selected materials and fabrication methods depend on the final application. These conductive textiles can be made by using conductive fibers or yarns, and coating fabric with conductive polymers or inks [7,8,9,10,11]. There are several available techniques that integrate conductive fibers or yarns into textile structures, such as knitting [12], weaving [13], embroidering [14], and sewing [15].

Conductive polymers are defined as organic polymers exhibiting a conductive or semi-conductive behavior. They combine some of the mechanical features of polymers with the electrical properties typical for metals [16,17]. They can be used to coat yarn surfaces or textile substrates by in situ polymerization [18], coating core yarns with a conductive Sheath [19], electrochemical deposition [20], and chemical vapor deposition [14,21]. Printing technologies can also be used to print conductive materials onto textile substrates using printing technologies such as screen printing, rotary printing, and inkjet printing [22,23,24,25]. Conductive patterns are created in the predetermined area instead of coating the entire textile surface. Inkjet printing offers lower deposition of droplets [26]. It also offers greater design versatility and production flexibility; however, it is limited by the viscosity and surface tension requirements of the conductive inks used, and the need of their high purity in solution form [27]. Printing with high-viscosity materials, like organic dielectrics, and dispersed particles, like inorganic metal inks, could provoke the problem of clogging in the nozzle [28].

Recently, several articles have been published in the field of textile-based electrodes used for ECG monitoring in the real time. Fong & Chung [29] developed a capacitive electrocardiograph (cECG) technique using a non-invasive ECG monitoring. It does not require direct contact between sensors and skin. However, this system poses several challenges and drawbacks, due to the fact that the coupling between the skin and sensor electrodes is weak. Moreover, as this system has been integrated to a chair, the subject had to be seated on it. As consequence, it was possible to move for subjects during monitoring. Also, because there was no direct physical contact between the subject and any grounding point, there was no discharge path for the electrostatic charge. Therefore, this electrostatic charge build-up could temporarily contaminate the ECG signal by adding noise. A stabilization period is required for the measurement of a clean and stable EC signal at low humidity. This problem has been partially solved by the design of hygroscopic fabric electrode with embedded polymer (FEEP). The principle of FEEP as a conductive electrode is intended to associate the humidity with the capacitive coupling, and to allow a stable and clear biomedical signal.

Weder et al. [30] have developed an embroidered textile electrode from polyethylene terephthalate yarn plasma coated with silver and ultra-thin titanium layer on top for passivation. Those electrodes were embedded into a breast belt. However, they had to be moisturized with a very low amount of water vapor from an integrated reservoir. In our opinion, this moisturizing will be an issue for long-term use, because the reservoir has to be filled up regularly. The advantage of this approach is that the monitoring is possible at rest, as well as when the subject is moving.

Yapici, & Alkhidir [31], worked on a fully-wearable medical garment for mobile monitoring of ECG signals from the wrists or the neck with minimal restrictions to regular clothing habits. Their approach is based on elastic bands with graphene-functionalized, textile electrodes and battery-powered, low-cost electronics for signal acquisition and wireless transmission. Textile-based dry electrodes were prepared by dip-coating nylon fabric with a graphene oxide suspension arranged ahead of time by following the protocol for the modified Hummers’ method. Then, the dipped textile was thermally treated to allow conformal cladding of graphene oxide flakes on individual fibers. The cross-correlation assessment and spectrum analysis of the ECG signal acquired form the conventional Ag/AgCl electrodes and from the textile electrodes showed the high quality of the developed system.

Majumder, S., et al. [32], published a comprehensive review of existing available sensors. In their review, many different approaches for textile-integrated electrodes have been mentioned, while launderability was just briefly discussed.

Although several prototypes of textile-based ECG electrodes with micro-controller-based acquisition and communication systems have been developed, their washability has not been analyzed and assessed. Unfortunately, all those prototypes are not ready for the market because textile structures used as substrates have to be washed. This is particularly true for underwear. At least fifty washing cycles must be guaranteed; otherwise nobody will buy an ECG monitoring system for personal use. In our opinion, the reliability and washability of those systems are the key issues that have to be investigated in order to make them ready for the market. This is the reason why our research and development are strongly focused on textile-based, fully-integrated ECG monitoring systems, and their capacity to be laundered and to provide reliable and safe use over the course of their lifetimes. Economic issues such as the cost of the used materials were also of concern to us.

Screen printing is cheap, and covers a huge range of manufactured items compared to inkjet printing. Conducting layers are produced from a viscous paste through a patterned fabric screen. In this article, the screen-printing process is selected because of its economic interest and relatively simple process to produce flexible textile electrodes. Poly(3,4-ethylenedioxythiophene) polystyrene sulfonate (PEDOT:PSS) is chosen as the conductive polymer, because it presents many attractive properties, such as excellent electrical properties, environmental stability, and decent biocompatibility [6].

In our previous research [33], a method for designing and production of PEDOT:PSS-based textile ECG electrodes has been announced; they have since been tested before and after 50 washing cycles. Data acquisition was realized using a portable an ambulatory device, the “Colson CardiPocket 2” (Cuers, France), which was able to record only analog signals. Therefore, it was impossible to analyze the spectrum and SNR of the acquired signal. Moreover, in our previous research, the ECG signal was not analyzed and validated by a cardiologist expert.

So far, wearable electronics have had limited commercial success, due to washability issues. This study is consequently devoted to (i) the development of washable, screen-printed cotton knitted electrodes based on PEDOT:PSS coating, (ii) the comparison of these coated electrodes with silver-plated ones, (iii) morphological characterization after washing, (iv) the measurement and analysis of ECG signals by a portable medical device and also by a low-cost Arduino-based open source device with great comfort for the user, and which is able to generate digital signals which are adapted to data treatment and different spectrum analysis.

## 2. Materials and Methods

For this investigation, 100% cotton single jersey and cotton/Lycra (95% cotton and 5% Lycra) single jersey (Mondial Tissu, Aix-en-Provence, France) samples were used. The choice of cotton fabric is mainly related to the advantages of cotton fibers, rather than their environmental benefits, as they are natural; they are soft, comfortable, breathable, and absorbent. They can stand up to abrasion wear and high temperatures. To achieve the best stretchability of the fabric, cotton is blended with Lycra.

Poly(3,4-ethylenedioxythiophene):poly(styrene sulfonate) type Clevios was purchased from Heraeus Conductive Polymers Division (Hanau, Germany). This commercial PEDOT:PSS product had to be chemically modified in order to make it suitable for textile fabrics for the screen-printing process, particularly to guarantee optimal washing behavior [33]. The nature of the chemical modification cannot be revealed in this article because of confidentiality issues [33]. However, it is possible to reveal that the viscosity of the compound has been modified, its adhesion capacity has been enhanced in order to better stand the washing process, and the compound has also been made compatible with textile substrates to avoid chemical damage.

**Figure 1:**
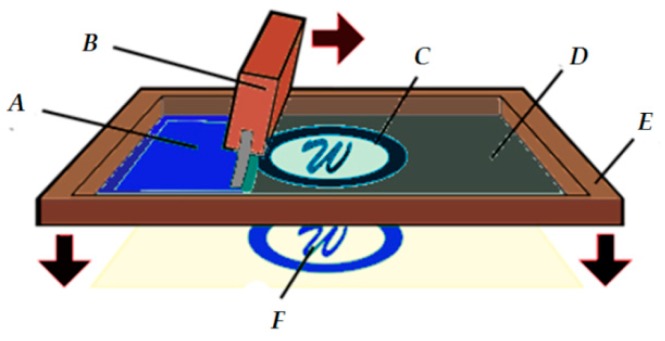
Screen printing process (A: PEDOT:PSS; B: squeegee; C: opening in screen; D: screen; E: screen frame, F: printed electrode).

**Figure 2:**
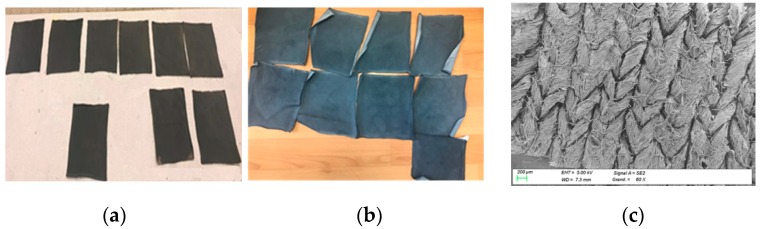
(**a**) cotton/Lycra; (**b**) 100% cotton single jersey electrodes coated by PEDOT:PSS. (**c**) SEM image for cotton single jersey electrode coated by PEDOT:PSS.

For both 100% cotton and cotton/Lycra, nine electrode samples with dimensions of 170 × 150 mm^2^ were manufactured by screen-printing (Figure 1) with a modified PEDOT:PSS solution, and then dried at 100 °C for 20 min (Figure 2). After drying, electrodes were rinsed in distilled water to eliminate all remaining particles.

The determination of the thickness of textile fabrics was performed according to ISO 5084:1996 standards [34], using a Sylvac machine (SODEMAT, Troyes, France).

Commercially-available, silver-plated textile electrodes (Innovative Textiles, Florence, Italy) were used in comparisons with our novel cotton electrodes.

The experimental electrical characterization was realized according to ASTM D 257-99 [35] and IEC 61340-5-1 Standards [36]. Surface resistivity is defined as the electrical resistance of the surface of the material. It is measured from electrode to electrode along the surface of the sample. As in our previous work [33], surface resistivity measurements were carried out by a KEITHLEY 8009 resistivity test device. The sample was placed between two concentric ring electrodes. Its resistance was measured by sourcing a known voltage, and measuring the resulting current using Ohm’s Law. From the resistance measurement, the resistivity was determined based on the physical dimensions of the test sample (Equation (1)).

(1) $$\rho s=53.4\frac{V}{I}\;$$

where ρs is the surface resistivity of the sample, *V* is the applied voltage from the electrometer, and *I* is the current read from the Ampermeter [33].

According to our previous work [33], cotton textile electrodes based on a modified PEDOT:PSS solution were washable, and provided decent ECG signals after 50 washing cycles in a normalized machine and using normalized detergent. Developed electrodes are supposed to reach the market in a wearable configuration for long-term monitoring. Therefore, tests in a domestic washing machine with commercial detergent were performed.

Before washing, textile electrodes were sewn onto a textile substrate, as they would be in the final product. The washing process was carried out with a commercial detergent (X.TRA Total, Roubaix, France) in a domestic washing machine (Miele, Paris, France). Each washing cycle comprised 35 min at 40 °C with 30 mL of detergent and a total load machine of 2.5 kg. The drying spinning speed was 600 rpm.

**Figure 3:**
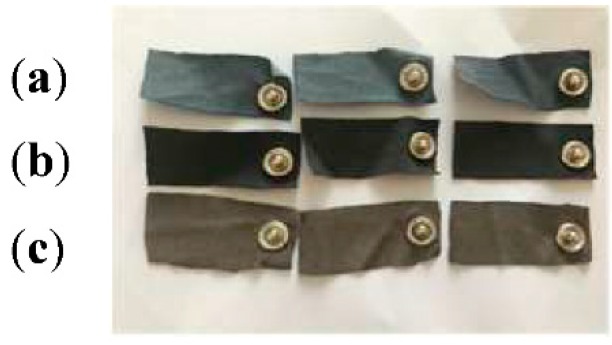
The shape of textile electrode used for ECG measurements; (**a**) 100% cotton; (**b**) cotton/Lycra; (**c**) silver-plated.

Screen-printed electrodes (cotton and cotton/Lycra knitted fabric) and silver-plated textile electrodes were cut into 50 × 20 mm^2^ pieces (Figure 3). Snap buttons were added in order to connect electrodes to electrocardiograph devices.

ECG measurements were performed by a portable medical device, “Colson CardiPocket 2”, and also by a SHIELD-EKG-EMG card from OLIMEX. The OLIMEX card was configured using an Arduino, and data analysis was processed using MATLAB (R2013a). Signals were filtered by a Butterworth passband filter (0.05–100 Hz) and Notch filter at 50 Hz to remove, respectively, motion artifacts and power line noises.

Three textile electrodes were placed on the right and left forearms, and a ground electrode is placed on the right ankle of a healthy female subject. Plastic clamps were applied to maintain electrodes on the skin in order to simulate the pressure that will be obtained with the aforementioned elastic structures. The recording was carried out for around 40 s with the subject sitting to avoid motion artifacts. Measurements were carried out without any skin preparation at the electrodes sites, and performed immediately after installing the electrodes.

The signal recorded is Lead I, corresponding to the voltage between the left arm (LA) electrode and right arm (RA) electrode. The ECG analyses were validated by an experienced cardiologist (Cardiology department, Pays D’Aix Hospital, Aix-en-Provence, France).

## 3. Results and Discussion

### 3.1. PEDOT:PSS Absorption

The weight percentages (wt.%) of PEDOT:PSS absorbed after drying are shown in Table 1. The PEDOT:PSS contains very little solid content dispersed in water. Its absorption capacity by textile fabrics depends on the intrinsic nature of the fibers. As cotton fiber is hydrophilic, water tends to be retained in the cotton. On the other hand, the Lycra is hydrophobic, meaning that its surface has few bonding sites for water molecules. Based on these explanations, the fact that cotton/Lycra, contains 5% Lycra decreases its capacity to absorb more PEDOT:PSS (6.3%) compared to 100% cotton electrodes, which absorb 7.4% of PEDOT:PSS.

### 3.2. Electrical Characterization

**Figure 4:**
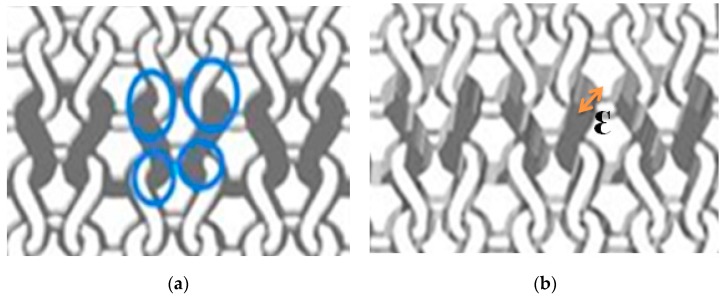
Stitch pattern of (**a**) thinner and (**b**) thicker single jersey fabric (contact between loops noted by blue circles).

To evaluate the electrical behavior of developed screen-printed cotton electrodes, surface resistivity was measured and values are given in Table 2. Typically, surface resistivity is inversely proportional to the wt.% of PEDOT:PSS absorbed. However, even though the 100% cotton electrode had more PEDOT:PSS than the cotton/Lycra electrode, the surface resistivity was higher for 100% cotton (117.3 kΩ) than that for cotton/Lycra (90.5 kΩ). In fact, the overall electrical resistance is not only related to the quantity of PEDOT:PSS absorbed, but also to the fabric thickness, as shown in Table 1 (725 µm for cotton/Lycra fabric versus 831 µm for 100% cotton fabric). To understand the electrical behavior related to the thickness of the textile fabric, Figure 4 shows the stitch pattern of a single jersey fabric. Generally, a knitted fabric is described as comprising interconnections of loops, with each loop (the basic unit of knitting [37]) consisting of a head, two legs, and two sinker loops joining the adjacent loops [38]. Thereby, there are four contact areas between the loop under consideration and the adjacent loops, as illustrated by blue circles in Figure 4a. Thinner cotton knitted fabrics tend to get wet and have good moisture transportation of PEDOT:PSS through the four-contact area between loops. For this reason, cotton/Lycra has lower surface resistivity than 100% cotton. The fabric thickness might play a more dominant role in the homogenous diffusion of PEDOT:PSS, and also in decreasing the surface resistivity of the electrode. Table 2 shows also that developed electrodes have a surface resistivity that is four to five times higher than that of silver-plated electrodes. The difference can be explained by the electric properties of material (conductive polymer and metal). However, it is not possible to establish a direct relation between the surface resistivity and the skin, i.e., the electrode contact impedance that is the most important for proper ECG signal recording.

In order to obtain a surface resistivity that is close to that of silver-plated electrodes, the wt.% of modified PEDOT:PSS was increased progressively without exceeding the limit absorption of the fabric to avoid wasting PEDOT:PSS, and also to increase the stiffness of the electrodes after drying.

As can be seen from Table 3, increasing wt.% of PEDOT:PSS implies a decrease of the surface resistivity of textile electrodes. For the cotton/Lycra electrode, the addition of 5.6 wt.%PEDOT:PSS decreases the surface resistivity by 52.2 kΩ (90.5 KΩ for 6.3 wt.% and 38.3 KΩ for 11.9 wt.% PEDOT:PSS). In contrast, for electrodes with pure cotton, surface resistivity decreases by 94.6 kΩ when adding 5.4 wt.% of PEDOT:PSS (117.3 KΩ for 7.4 wt.% and 22.7 KΩ for 12.8 wt.% PEDOT:PSS). This difference can be attributed to the different thickness of the knitted fabric. As 100% cotton fabric has high thickness compared to cotton/Lycra fabric, the addition of wt.% PEDOT:PSS was helpful to enhance the PEDOT:PSS absorption by the capillary action of cotton fibers, which increases the electrical interaction between fibers, and hence between loops of the knitted fabric. Table 3 also includes the results of a very low surface resistivity of 22.7 kΩ for 100% cotton electrodes containing 12.8 wt.% of PEDOT:PSS, which exhibited surface resistivity close to that of silver-plated electrodes.

**Figure 5:**
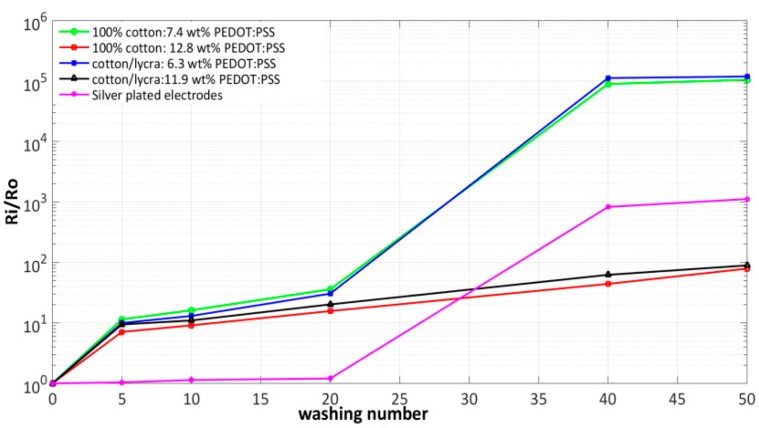
Variation of the ratio Ri/Ro vs washing cycles for PEDOT:PSS and silver-plated electrodes.

Figure 5 presents variations of the ratio Ri/Ro during 50 washing cycles for both PEDOT:PSS and silver-plated electrodes, where Ro is the surface resistance before washing and Ri is the surface resistance after the i-th washing cycle. For all electrodes, the resistance increases linearly as a function of the number of washing cycles, but with different slopes. For PEDOT:PSS-coated electrodes, Ri/Ro increases similarly until 5 washing cycles. From 1 to 20 washing cycles, the resistance ratio (Ri/Ro) is respectively equal to 20 and 30 for cotton/Lycra electrodes with 11.9 wt.% and 6.3 wt.% PEDOT:PSS. Also, this ratio is respectively equal to 16 and 36 for pure cotton electrode containing 12.8 wt.% and 7.4 wt.% PEDOT:PSS. However, the silver-plated electrodes had a very slight increase in resistance ratio (Ri/Ro) equal to 1.2. From 20 to 40 washing cycles, higher slopes were noticed for silver-plated electrodes and also for 6.3 wt.% and 7.4 wt.% PEDOT:PSS pure cotton electrodes. After 40 washing cycles, the resistance ratio Ri/Ro increased slightly for all electrodes. To summarize, after 50 washing cycles, the ratio Ri/Ro increased by 5 orders magnitude for 6.3 wt.% and 7.4 wt.% PEDOT:PSS electrodes, 3 orders magnitude for silver-plated electrodes, and by 1 order magnitude for electrodes with high amounts of PEDOT:PSS (11.9 wt.% and 12.8 wt.% PEDOT:PSS). It can be inferred here that adding 5.6 wt.% PEDOT:PSS to cotton/Lycra and 5.4 wt.% PEDOT:PSS to pure cotton decreases the resistance ratio Ri/Ro by 4 orders magnitude after 50 washing cycles.

**Figure 6:**
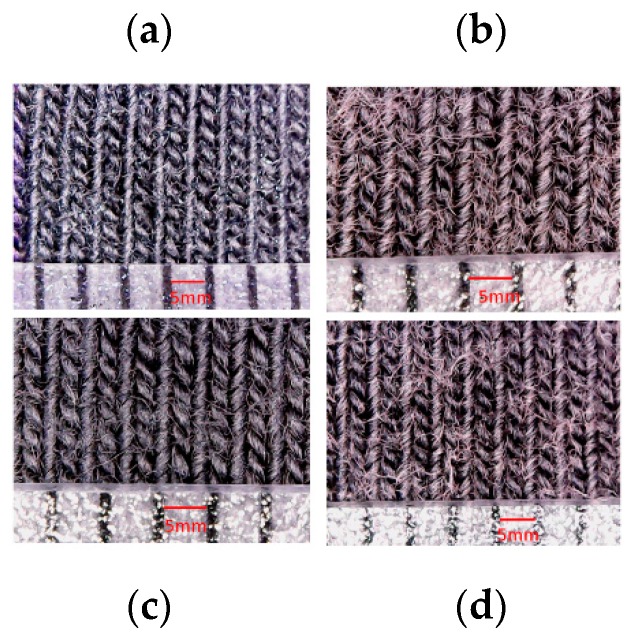
Surface morphology of cotton/lycra electrodes: (**a**) before washing: 6.3 wt.% PEDOT:PSS; (**b**) after 50 washes: 6.3 wt.% PEDOT:PSS; (**c**) before washing: 11.9 wt.% PEDOT:PSS; (**d**) after 50 washes: 11.9 wt.% PEDOT:PSS.

**Figure 7:**
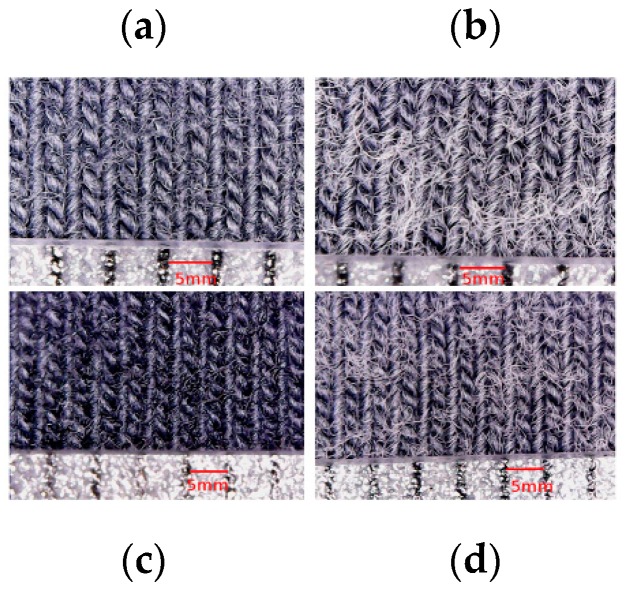
Surface morphology of pure cotton electrodes: (**a**) before washing: 7.4 wt.% PEDOT:PSS; (**b**) after 50 washes: 7.4 wt.% PEDOT:PSS; (**c**) before washing: 12.8 wt.% PEDOT:PSS; (**d**) after 50 washes: 12.8 wt.% PEDOT:PSS.

**Figure 8:**
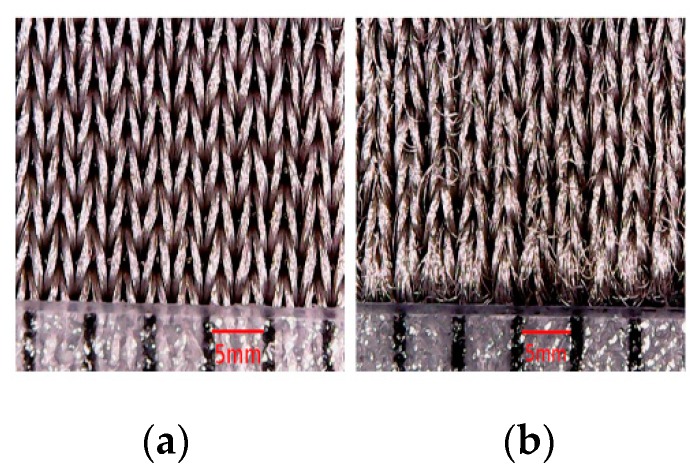
Surface morphology of silver-plated electrodes (**a**) before washing; (**b**) after 50 washing cycles.

In general, this difference in variation of electrical resistance after washing can be explained by the fact that the remaining quantity of PEDOT:PSS after each washing cycle is higher for electrodes with high wt.% PEDOT:PSS. In fact, mechanical movements (friction, bending, etc.) in the washing machine significantly affected loop shapes, which has a tendency to create pills (Figure 6, Figure 7 and Figure 8). Repeated washing cycles increase the degree of damages to fibers, producing small cracks and fractures (Figure 8b), and therefore, leading to adhesion failures between knitted fabric and PEDOT:PSS coating, and also of silver coating for silver-plated electrodes. Based on this result, it can be concluded that high wt.% PEDOT:PSS enhances the conductivity of textile electrodes, and as a result, pure cotton electrodes with 12.8 wt.% PEDOT:PSS are a better choice in terms of electrical resistance.

### 3.3. Electrocardiography Analysis

**Figure 9:**
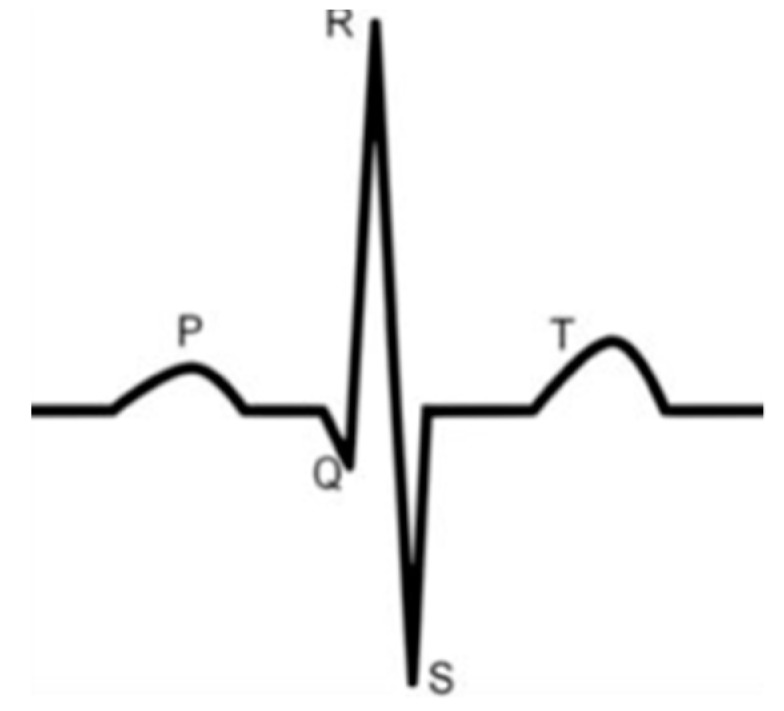
General waveform of an electrocardiogram (ECG).

In order to determine the best electrodes to obtain a high quality signal, i.e., a signal without any missing P, Q, R, S, and T waves (Figure 9), all electrodes were evaluated after repetitive washing by recording the ECG signals.

#### 3.3.1. ECG Recorded by Portable Medical Device

**Figure 10:**
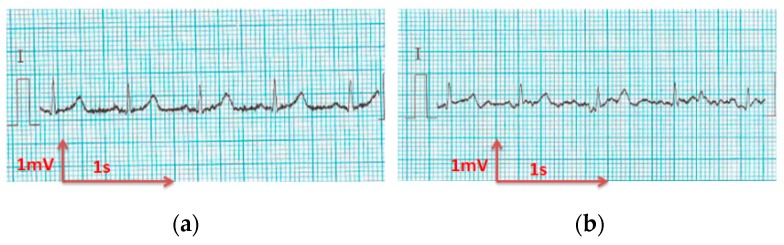
ECG signal obtained by 100% cotton electrodes with 7.4 wt.% PEDOT:PSS: (**a**) before washing; (**b**) after 50 washing cycles.

**Figure 11:**
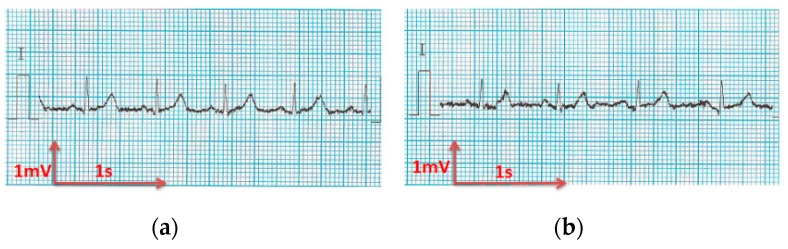
ECG signal obtained by 100% cotton electrodes with 12.8 wt.% PEDOT:PSS: (**a**) before washing; (**b**) after 50 washing cycles.

**Figure 12:**
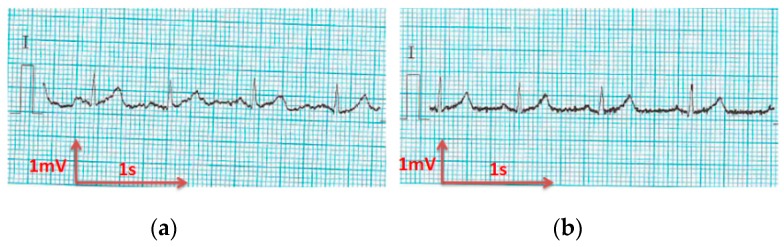
ECG signal obtained by cotton/Lycra electrodes with 6.3 wt.% PEDOT:PSS; (**a**) before washing; (**b**) after 50 washing cycles.

**Figure 13:**
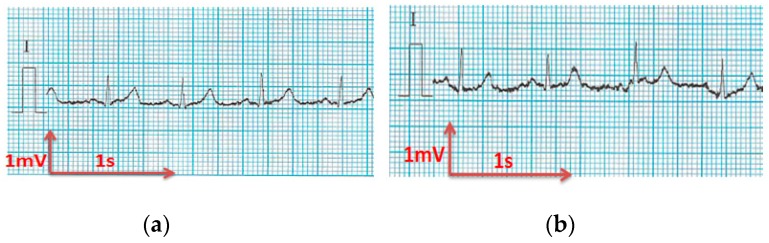
ECG signal obtained by cotton/Lycra electrodes with 11.9 wt.% PEDOT:PSS: (**a**) before washing; (**b**) after 50 washing cycles.

**Figure 14:**
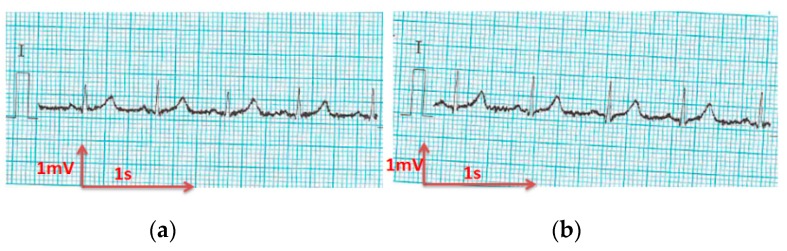
ECG signal obtained by silver-plated electrodes: (**a**) before washing; (**b**) after 50 washing cycles.

All electrocardiograms (Figure 10, Figure 11, Figure 12, Figure 13 and Figure 14) recorded by the portable medical device have stable P, R, and T wave amplitudes, both before or after 50 washing cycles. P wave (corresponding to atrial depolarization), QRS complex (corresponding to ventricle depolarization), and T wave (corresponding to the repolarization of the ventricle) were all detectable. Since the signal is drawn in the paper from a portable medical device, we can only observe the quality of signals by visual inspection. It is easy to interpret the rhythm of the ECG. P waves are clear and the PP intervals are distinct. The PR intervals (190 ms) confirm that there is no AV bloc. It is a normal sinusoidal rhythm, so the cardiologist could eliminate atrial fibrillation, the most common effect of cardiac rhythm, or symptom of some bigger problem.

The corrected QT interval is under 400 ms, and the ST-T segment is at the isoelectric line, implying no argument for silent ischemia. Thus, the acquired signals are acceptable and suitable for long-term monitoring of the rhythm according to our experienced cardiologist.

#### 3.3.2. ECG Recorded by Low-Cost Arduino Based Open Source

**Figure 15:**
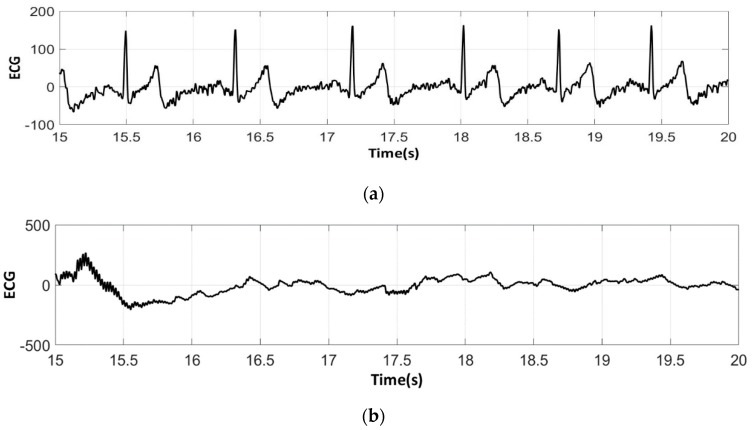
ECG signal obtained by 100% cotton electrodes with 7.4 wt.% PEDOT:PSS: (**a**) before washing; (**b**) after 50 washing cycles.

**Figure 16:**
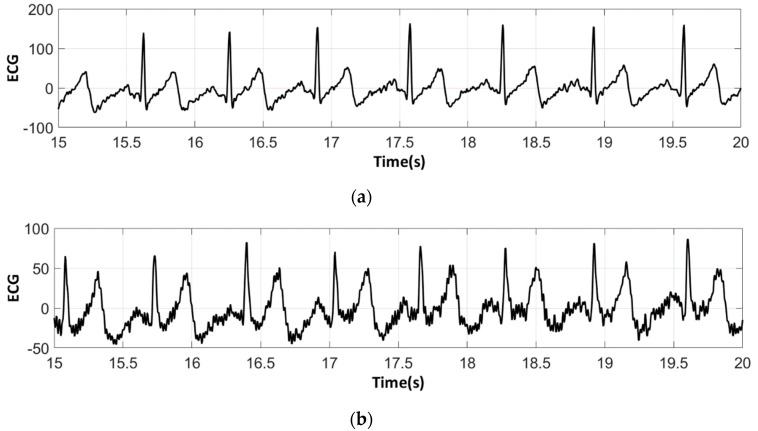
ECG signal obtained by 100% cotton electrodes with 12.8 wt.% PEDOT:PSS: (**a**) before washing; (**b**) after 50 washing cycles.

**Figure 17:**
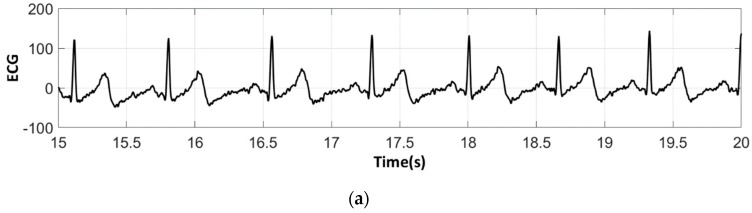

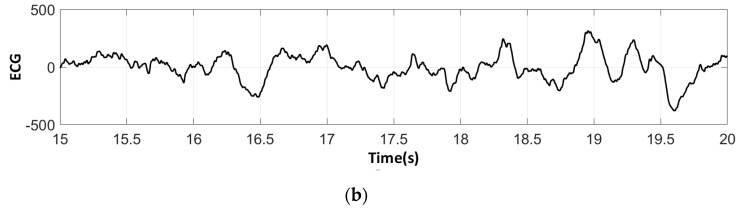
ECG signal obtained by cotton/Lycra electrodes with 6.3 wt.% PEDOT:PSS: (**a**) before washing; (**b**) after 50 washing cycles.

**Figure 18:**
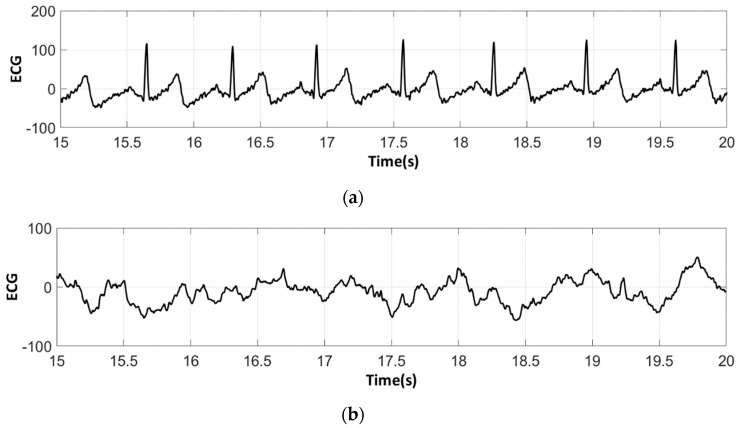
ECG signal obtained by cotton/Lycra electrodes with 11.9 wt.% PEDOT:PSS: (**a**) before washing; (**b**) after 50 washing cycles.

**Figure 19:**
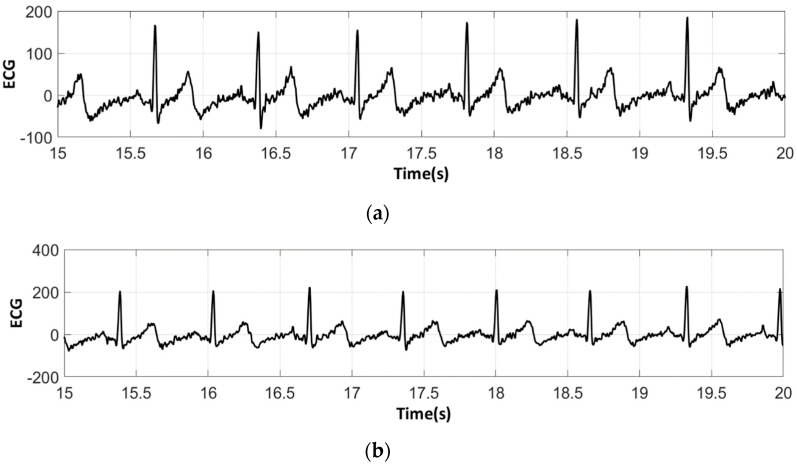
ECG signal obtained by silver-plated electrodes: (**a**) before washing; (**b**) after 50 washing cycles.

The analysis of the electrocardiograms recorded by the low-cost, Arduino-based open source device showed that increasing wt.% PEDOT:PSS improved strongly the quality of ECG signals. In fact, the signal recorded by 100% cotton electrodes before washing provided more noise when using low quantity of PEDOT:PSS (7.4 wt.%), (Figure 15a) compared to electrodes with 12.8 wt.% PEDOT:PSS. After 50 washing cycles, unclear waveforms P, QRS, and T waves for electrodes with 7.4 wt.% PEDOT:PSS (Figure 15b) appeared. In spite of the repetitive washing of electrodes with 12.8 wt.% PEDOT:PSS, the recorded ECG still retained complete and stable P, QRS complex, and T waveforms (Figure 16). As seen in Figure 17 and Figure 18, regarding cotton/Lycra electrodes, before washing, there was no obvious difference in terms of the detectability of P, QRS complex, and T waves between 6.3 wt.% or 11.9 wt.% of PEDOT:PSS. However, after 50 washing cycles, electrocardiographic waves were not detectable. Concerning silver-plated electrodes, P and T waves were a bit polluted by noise, before and after washing, but were still detectable (Figure 19).

Since all electrocardiographic P, T waves, and QRS complex were identifiable by the portable medical device, the serious attenuation, noise, and distortion of ECG signals recorded by the low-cost, Arduino-based, open source device could be explained by the fact that the device used does not contain the compatible recording amplifiers which compensate for the high contact impedance between textile electrodes and skin. In fact, ECG recordings were carried out without hydrating the skin, but only based on natural skin moisture and perspiration. The high contact impedance is related to the low mobility of ions across the highly-resistant stratum corneum layer of the skin, which causes weak conductivity between the electrodes and the skin. This can be seen in ECGs acquired with the Arduino device with which, on top of having some noise, one can see a kind of biphasic T-wave which is in fact an artifact coming from the Arduino analog chain.

SNR ratios for different electrodes are given in the following table (Table 4). It is important to note that they are computed, even for signals with corrupted ECG signals.

**Figure 20:**
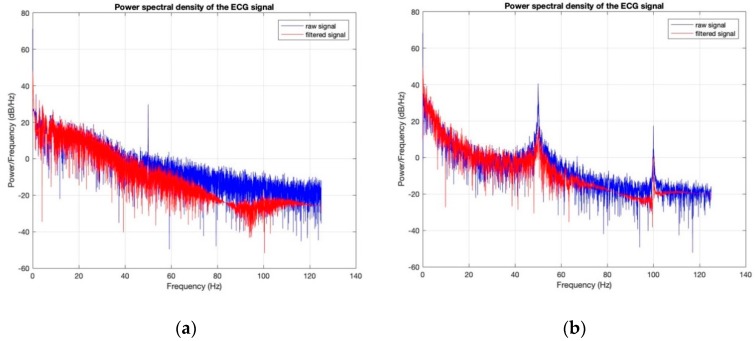
Power Spectral Density of ECG signal obtained by 100% cotton electrodes with 7.4 wt.% PEDOT:PSS: (**a**) before washing; (**b**) after 50 washing cycles.

**Figure 21:**
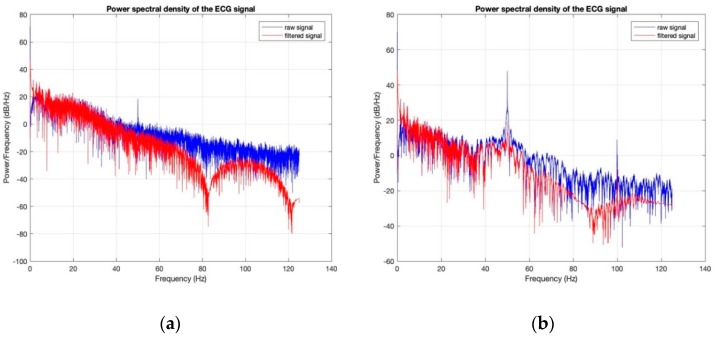
Power Spectral Density of ECG signal obtained by 100% cotton electrodes with 12.8 wt.% PEDOT:PSS: (**a**) before washing; (**b**) after 50 washing cycles.

**Figure 22:**
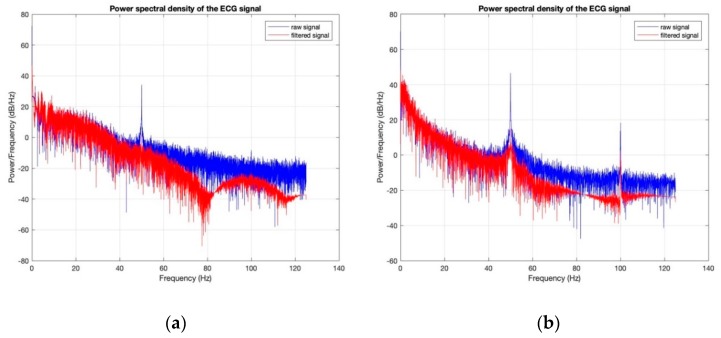
Power Spectral Density of ECG signal obtained by cotton/Lycra electrodes with 6.3 wt.% PEDOT:PSS: (**a**) before washing; (**b**) after 50 washing cycles.

**Figure 23:**
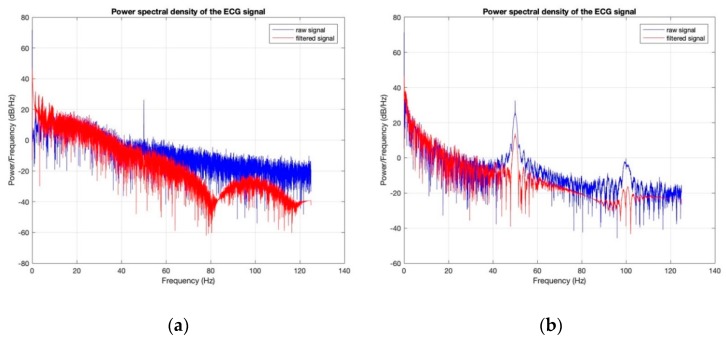
Power Spectral Density of ECG signal obtained by cotton/Lycra electrodes with 11.9 wt.% PEDOT:PSS: (**a**) before washing; (**b**) after 50 washing cycles.

**Figure 24:**
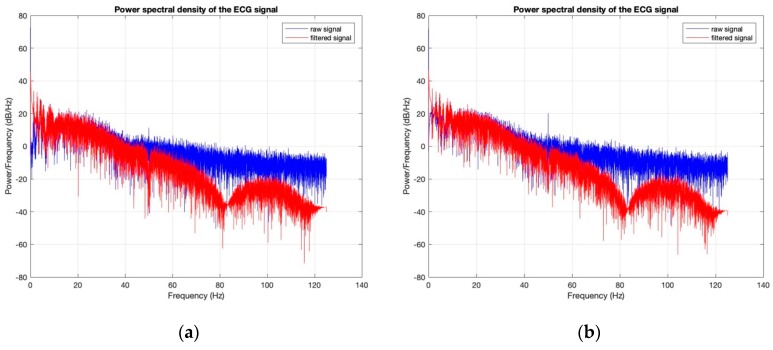
Power Spectral Density of ECG signal obtained by silver-plated electrodes: (**a**) before washing; (**b**) after 50 washing cycles.

Power spectral densities of all the ECG signals before (raw signal) and after filtering (Figure 20, Figure 21, Figure 22, Figure 23 and Figure 24) show clearly that 50 Hz noise is removed. Moreover, it is possible to see that the ECG signals contain frequencies up to approximately 40 Hz. The sampling frequency used with Arduino kit was set to 250 Hz.

**Figure 25:**
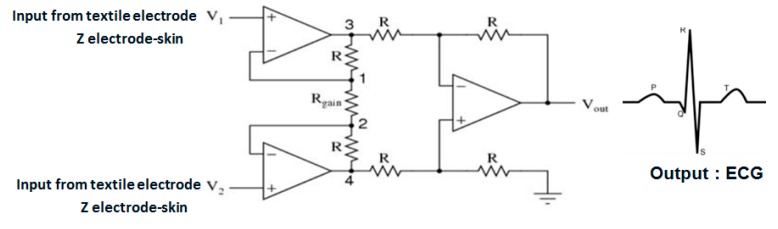
General circuit for ECG acquiring.

Contact between the skin and textile electrodes is an important and delicate issue in the development of wearable ECG device. Therefore, our ECG acquisition procedure was carried out on a sitting subject in order to avoid bad contact due to the movement of subject. However, this problem should be studied and overcome. Hence, our future studies will be focused on the determination of contact impedance after washing, and subsequently, the design will be undertaken of the proper amplifiers (Figure 25) for the flexible electronic module which will be connected to our developed textile electrodes and fully embedded into garment for long-term ECG monitoring.

## 4. Conclusions

In this study, pure cotton and cotton/Lycra knitted fabrics were used to make flexible textile electrodes by a screen-printing process. Screen printing was selected because it is fast, more economical, and covers a wide range of manufactured items. Poly(3,4-ethylenedioxythiophene) polystyrene sulfonate (PEDOT:PSS) is chosen as a conducting polymer because of its high conductivity, environmental stability, and decent biocompatibility. Textile electrodes have good air permeability, perspiration absorption, and quick dry property, promoting the comfort for the users.

- The thickness of the fabric plays a dominant role in increasing the surface resistivity while using small amount of PEDOT:PSS. The thinner the fabric, the better the distribution of PEDOT:PSS coating will be.
- In this work, silver-plated electrodes were also used to compare their performance with our developed PEDOT:PSS textile electrodes. The conductivity of cotton textile electrodes was enhanced by increasing the amount of PEDOT:PSS coating, which decrease the surface resistivity until obtaining closed value to that of silver electrodes. Subsequently, the effects of repetitive washing process on electrical properties, morphology and ECG quality of textile electrodes were also investigated. After 50 washing cycles, the ratio Ri/Ro increased by 5 orders magnitude for 6.3 wt.% PEDOT:PSS pure cotton electrode and 7.4 wt.% PEDOT:PSS cotton/Lycra electrode, 3 orders magnitude for silver-plated electrodes and by 1 order magnitude for electrodes with high amounts of PEDOT:PSS (11.9 wt.% on cotton/Lycra electrodes and 12.8 wt.% on pure cotton). Results reveal that adding 5.6 wt.% PEDOT:PSS to cotton/Lycra and 5.4 wt.% PEDOT:PSS to pure cotton decreases the resistance ratio Ri/Ro by 4 orders magnitude after 50 washing cycles. Consequently, pure cotton electrodes with 12.8 wt.% PEDOT:PSS are the best choice from the viewpoint of the electrical resistance.
- Mechanical stresses during the washing process affect the surface morphology of textile electrodes. They lead to the entanglements of loose fibers that form roughly spherical accumulation of fibers called “pills”, that are distinct from the electrode surface.
- For all textile electrodes, ECG signals were recorded by a portable medical device as well as with a low-cost, Arduino-based open source device. Using portable medical devices, all electrocardiographic P, T waves, and QRS complexes were identifiable, despite higher contact impedance of PEDOT:PSS textile electrodes. Signal quality analysis by cardiologist showed that these textile electrodes, used in ambulatory conditions for heart monitoring, allow the detection of rhythmic disorders (Atrial Fibrillation, ventricular tachycardia, etc.) and conduction troubles (sinus dysfunction, atrioventricular block etc.), and can detect myocardial ischemia (ST segment underlining) in some cases. Such a system, integrated into garments, can be used in real-time and in continuous mode to monitor the user’s heart in a comfortable way, and to detect possible issues that, with additional analysis if needed, could be avoided, such as strokes. Regarding low-cost devices, assuming that high contact impedance between the skin and textile electrodes is the cause of no detectability or noise and severe distortion of cardiac waveforms, it was concluded that the amplifiers of the low-cost device are not appropriate to record ECG of our developed PEDOT:PSS textile electrodes, and also, that of commercial silver-plated electrodes. Textile electrodes inherently provide high contact skin-electrode impedance because the ECG recording is based only on natural skin moisture and perspiration and without any added gel. Therefore, compatible recording amplifiers with high input impedance are required to compensate for the high contact impedance skin-electrode, which is related to the low mobility of ions across the highly-resistant stratum corneum layer of the skin that causes weak conductivity between the electrodes and the skin.
- The authors consider that the pure cotton electrodes could be embedded into garments for long-term ECG monitoring.

## Figures and Tables

**Table 1:** Thickness of textile substrate before coating and wt.% of PEDOT:PSS absorbed after drying.

	Thickness of Textile Substrate before Coating (µm)		wt.% of PEDOT:PSS Absorbed after Drying	
Textile electrodes	Avg	St Dev	Avg	ST Dev
100% cotton	831	15.6	7.4	0.1
95% Cotton 5% Lycra	725	4.9	6.3	0.2

**Table 2:** Surface resistivity of textile electrodes.

Textiles Electrodes	Surface Resistivity before Washing (kΩ)	Standard Deviation (kΩ)
100% cotton	117.3	0.217
95% Cotton 5% Lycra	90.5	0.275
Silver-plated electrode	21.4	0.035

**Table 3:** Comparison of surface resistivity of textile electrodes after increasing wt.% PEDOT:PSS.

	Surface Resistivity (kΩ)	Standard Deviation (kΩ)
100% cotton: 7.4 wt.% PEDOT:PSS	117.30	0.21
100% cotton: 12.8 wt.% PEDOT:PSS	22.70	0.04
Cotton/Lycra: 6.3 wt.% PEDOT:PSS	90.50	0.27
Cotton/Lycra: 11.9 wt.% PEDOT:PSS	38.30	0.04
Silver-plated electrodes	21.40	0.03

**Table 4:** Signal to-noise ratio (SNR) of textile electrodes before and after washing.

	SNR (dB) before Washing	SNR (dB) after Washing
100% cotton: 7.4 wt.% PEDOT:PSS	24.6311	11.8333
100% cotton: 12.8 wt.% PEDOT:PSS	33.0505	7.6069
Cotton/Lycra: 6.3 wt.% PEDOT:PSS	17.8022	11.5040
Cotton/Lycra: 11.9 wt.% PEDOT:PSS	27.7690	15.6060
Silver-plated electrodes	34.7203	33.1449
